# Annotations

**Published:** 1900-12-08

**Authors:** 


### Annotations.

### The Midwives Question at Manchester.

The great question whether it is best to mend the midwives or to end them, although it quietens down for a time, never quite dies out, and lately, notwithstanding that the country at large is somewhat tired of the whole affair, a considerable local agitation has arisen in Manchester over one of the side issues almost of necessity involved in it, namely, the propriety of medical men undertaking the work of teaching midwives. Now, we do not think it well at the present moment to discuss this problem in the form in which it has arisen in Manchester, where the discussion has assumed far too personal and individual a character to render it possible to arrive at any useful or generally applicable conclusion. At the same time we would point out that some of those who protest against instruction being given to midwives seem to beg the whole question, taking it for granted as a thing settled past dispute that for midwifery to be undertaken by midwives is wrong. The greater includes the less, and to those who regard the midwife as an evil the teaching of the midwife must appear evil also.

This, however, brings us round again at once to the main question: Are midwives to continue to attend women in their confinements?—a question which, we need hardly say, remains entirely unaffected by such a subordinate issue as that which is involved in the ethics of teaching them their work. There are no doubt many ethical difficulties in the way of teaching a subject which verges so nearly upon the domains of surgery as does that of normal midwifery, and we are not concerned to defend those who may have appeared to overstep the bounds of professional propriety in so doing. But when we find that the mere fact of giving such teaching is being held up as in itself an ethical impropriety we must protest. If the midwife is not to be abolished, it is clear that she must be taught her trade, and those who think she should be abolished, instead of railing at her teachers, ought as a first step to produce some workable scheme by which women living in outlying districts shall be provided with better and safer attendance in their labours than could be secured to them by taking care that the midwife shall be taught her work. Not till it is shown that it is possible and practicable to provide qualified medical attendance for every woman in her confinement will the question of abolishing the midwife be ripe even for discussion. Till then it seems only reasonable that the midwife should be instructed how to do her work in as safe a manner as possible, and we would submit that the only effect of all such attempts as are being made on one side and another to deter the medical officers of recognised hospitals and maternities from taking up the work of teaching midwives can only result in driving such teaching into the hands of competing private venture institutions—the very thing which is most to be deprecated.

### The Diploma in Public Health.

A considerable amount of time was devoted by the General Medical Council last week to a discussion of the terms on which the Diploma in Public Health should be granted. Evidently a certain amount of influence had been brought to bear on behalf of various Government Departments, Army, Navy, and Colonial, with the object of shortening the time of study required for obtaining these diplomas, and the Public Health Committee had formulated a scheme according to which the candidates would be divided into two classes, junior and senior, the latter of whom, being practitioners of ten years' standing, would be exempted from a large portion of the practical part of the curriculum demanded of the juniors. Sir John Tuke, in a most cogent speech, pointed out the evils of the proposed scheme, and showed that if the military or the colonial authorities wished the members of their Services to possess a diploma in public health, in desiring which they were much to be commended, the proper course was not to ask to have the standard of the curriculum lowered to suit their convenience, but for them to grant their men study leave so that they might not only have the diploma, but obtain the full training which the possession of such a diploma ought to connote. In the end the scheme was entirely modified and the proposed division of the candidates into two classes was very properly done away with. As was said by Mr. Glover in the face of recent experiences and calamities from enteric fever in South Africa, the present seems an unfortunate moment to reduce the character of the curriculum in the case of colonial candidates for a public health diploma. It was, he said, an entirely new departure for the Council to divide candidates into two classes. It might be good for the Colonial candidates, but it could not be good for the Colonies themselves.

### Electric Trams and County Boundaries.

The success of the Central London Railway seems at last to have opened the eyes of Londoners to the fact, patent enough in every other quarter, that it is to electricity that we must look for our chief means of locomotion in the future, and already we hear of untold millions which are to be spent in tunnelling the town in every direction for purposes of electric railways. All this is good so far as it tends to relieve our congested streets and to enable us to be carried quickly from one part of London to another without undergoing an "underground" suffocation. Regarding the matter, however, from a public health point of view one must admit that unless these new lines are supplemented by a large and complete system of suburban electric tramways they will fail to fulfil their highest mission, which is not merely to facilitate local transit but to widen out the city, and to scatter its population over a far larger area than it occupies at present. The attractiveness of town life largely arises from the comforts and conveniences with which the town-dweller is constantly surrounded, and among these conveniences facility for locomotion is one of the most important. Electric transit, then, unless extended far into the surrounding country, will but tend to forge the fetters all the firmer by which we are held fast to life in towns. It is a curious and an interesting thing, and one showing well how impossible it is to attain finality in any scheme of human government, that just at the moment when we have divided the county of London into a brave show of cities and boroughs, each with its mayor and corporation, and its semi-independent corporate existence, London should at last be on the very verge of obtaining a system of transit which may render the present boundaries of the county as meaningless and as obstructive as the boundaries of the City began to be two or three hundred years ago. With electric trams, electric railways, and electric distribution of power, it is hard to say what will set the limit to London's progress. This, however, is certain that under such conditions London will not be contained within the present limits of its county.

